# A Systematic Review of Community Pharmacy‐Led Depression Services: Service Components, Outcomes, and Implementation Barriers and Facilitators

**DOI:** 10.1155/da/9977874

**Published:** 2026-07-10

**Authors:** David Kernaghan, Hisham Alshammari, Gazala Akram, Judith Pratt, Margaret Maxwell, Margaret C. Watson, Natalie Weir

**Affiliations:** ^1^ School of Health in Social Sciences, University of Edinburgh, Doorway 6 Medical School, Teviot Place, Edinburgh, EH8 9AG, UK, ed.ac.uk; ^2^ Strathclyde Institute of Pharmacy and Biomedical Science, 161 Cathedral Street, Glasgow, G4 0RE, UK; ^3^ University of Stirling, Stirling, FK9 4LA, UK, stir.ac.uk

## Abstract

**Background:**

Depression is the most common mental ill health condition, and its prevalence is increasing. Despite this, its treatment is variable often due to a lack of capacity within healthcare systems to support this vulnerable population. Community pharmacy staff could offer additional support. This systematic review identifies depression services led by community pharmacy staff, their service components, outcomes, and barriers/facilitators to their implementation.

**Methods:**

Four bibliographic databases were searched (Medline, EMBASE, PsycINFO, and CINAHL) from 2000 onwards. Title/abstract and full‐text screening was conducted. Data on the service components were mapped to the Template for Intervention Description and Replication (TIDieR). Clinical, humanistic, economic, and service outcomes were charted. Barriers and facilitators were mapped to the Consolidated Framework for Implementation Research (CFIR). Quality assessment was performed using the Quality Assessment with Diverse Studies (QuADS) tool.

**Results:**

Fifty studies were included. Seventeen studies identified general attitudes regarding community pharmacy services for depression, which were generally supportive. The majority (*n* = 33) explored an implemented depression service focusing on depression advice/education (*n* = 15), screening (*n* = 12), medication adherence (*n* = 4), medication review (*n* = 1), and disease therapy management (DTM) (*n* = 1). Clinical outcomes were the most commonly reported types of outcomes, with varied results. Key facilitators were linked to the pharmacy ‘inner setting’, including accessibility of community pharmacies, the use of private consultation rooms, and skills/training of staff. Barriers to service delivery related often to the external ‘outer setting’, especially societal stigma, low public awareness of pharmacy roles, funding constraints, and limited collaboration with other healthcare professionals.

**Conclusion:**

This international review identified a range of different services that community pharmacy staff can deliver to support people with depression, ranging from supporting diagnosis, health literacy, and management plans. The accessibility of community pharmacies for depression service delivery warrants further investigation. However, limited empirical evidence of clinical and economic outcomes and reported implementation barriers may complicate broader implementation.

## 1. Introduction

Depression is the most prevalent mental ill health condition, with global estimates suggesting more than 300 million people are affected [1]. The prevalence of depression and other common mental health disorders has increased over the past 2 decades [2]. Each year in England, one in five people will experience a common mental health condition [3]. In the United Kingdom, over two million people are on waiting lists for mental health support [4], and in Scotland, adults reporting two or more symptoms of depression increased to 11% in 2020 [5, 6]. Similar trends in prevalence are occurring worldwide [7, 8].

Community pharmacies are uniquely accessible, often with extended opening hours and greater accessibility when compared with other health providers in the primary care setting. In the United Kingdom, over 90% of the population live within 20 min of their local pharmacy [9, 10], and up to 70% of people visit a community pharmacy monthly [11]. Community pharmacies are also typically located within socially deprived areas [12], where people are twice as likely to experience mental ill health [13]. Additionally, research has identified a willingness of community pharmacy personnel to support people with mental ill health [14, 15], with scope to act as ‘front‐line advisers’ [15], and have ongoing rapport with patients [14]. However, implementation of such services could be complicated by lack of training [16–18], insufficient resources [16], time constraints [17, 19, 20], pharmacy personnel concern with ‘making the situation worse’ [16], and limited referral options [19].

The role of community pharmacy personnel has evolved to now offer enhanced clinical services [21]. This includes support for smoking cessation [22], alcohol/substance misuse [22], weight management [22], and advice, treatment, and referral services [10]. In the United Kingdom, community pharmacy personnel also supported well‐being needs by offering safe spaces for domestic abuse sufferers [23], indicating capacity for psychological and social crisis management. The UK’s Stepped Care Approach to mental health care identifies primary care as the key setting for identification, assessment, psychoeducation, monitoring, and referral, and an International Pharmaceutical Federation review identified that community pharmacists have a role in mental health triage, early detection, optimising medicines, adherence support, education, and collaborative care [24]. The Royal Pharmaceutical Society (RPS), the professional body for pharmacy in Great Britain, postulates that community pharmacy personnel can have a role in mental health by identifying vulnerable individuals, accelerating access to support, collaborating with the wider healthcare team, offering support to patients initiating antidepressants, and signposting/onward referral [25, 26].

There is a growing literature, mainly from global north areas, which demonstrate the capability and capacity of community pharmacy personnel in the support of people with depression [27–30]. Reviews published in 2011 [30] and 2012 [27] identified services to promote adherence to antidepressants. A more recent review [29] of depression screening services identified positive impacts such as treatment initiation. In 2021, a review of community pharmacy depression services involving educational interventions, telehealth, medication reviews, and mindfulness programmes identified improvements in medication adherence and stigma reduction [28]. However, these reviews have focused only on a single type of depression service (i.e. depression screening [29] or medication adherence [27, 30]), focused on a subset of patient‐reported outcomes [28], are no longer timely (>10 years ago) [27, 30], or included only randomised control trial design [31]. This systematic review consolidates the evidence on all types of community pharmacy–led depression services, focusing on (i) description of the implemented pharmacy–led depression services; (ii) clinical, humanistic, economic, and service outcomes; (iii) and the determinants of implementation success (i.e. barriers and facilitators).

## 2. Materials and Methods

This review adheres to the Preferred Reporting Items for Systematic reviews and Meta‐Analyses (PRISMA) 2020 checklist [32].

### 2.1. Search Strategy

The databases Medline, EMBASE, PsycINFO, and CINAHL were initially searched on 24/01/2023, with an updated search completed in September 2025. Search terms included synonyms of ‘community pharmacy’ and ‘depression services’ and utilised Boolean operators and truncation codes (Appendix 1: Table A1). Studies not published in English were translated using the online translation software Google Translate. Reference lists of included articles were searched. Unpublished or grey literature was identified through the medRxiv repository, the OpenGrey.eu database, and the RPS e‐Library. Authors who conducted earlier reviews of pharmacy mental health services were contacted to identify other potentially relevant literature, and calls for information were posted on social media. The protocol for this systematic review was published on PROSPERO (CRD42023397704).

### 2.2. Eligibility Criteria

Qualitative, quantitative, and mixed‐method study designs that reported empirical data, published from 2000 onwards, were eligible for inclusion. Books, editorials, lecture commentaries, and conference proceedings were excluded. Eligible studies explored the concept of community pharmacy–led depression services (e.g. studies exploring preimplementation perceptions and general attitudes) or reported on the implementation/evaluation of a community pharmacy–led depression service. Studies were excluded if they explored more generally the role expansion of community pharmacy staff (e.g. prescribing) or facets of communication/consulting (e.g. social prescribing and motivational interviewing), unless these were elements that were part of a defined service specific to supporting people with depression. Only studies relating to services for adult patients (≥18 years) were included.

### 2.3. Screening

Screening was facilitated by Covidence software. Independent duplicate screening was undertaken by two researchers (DK and NW) of 20% of titles and abstracts screening and achieved proportional agreement of 92% (excellent [16]), with a Cohen’s kappa of 0.51 (0.4–0.7, classified as ‘good’). The remaining abstracts were screened by one individual (DK) [17]. Independent duplicate screening was undertaken (DK and NW) of full‐text articles. Conflicts were resolved by discussion with a third team member (MM, MW, GA, or JP).

### 2.4. Data Extraction

A data extraction table was devised and piloted comprising (i) study characteristics (e.g. country of origin, year study conducted, and eligibility criteria of patients); (ii) depression service components; (iii) clinical, humanistic, economic, and service outcomes; and (iv) barriers and facilitators [33]. Duplicate data extraction was undertaken by two researchers (DK and NW) of 20% of studies; the results were discussed and confirmed the quality and accuracy of the data extraction before the remaining data were extracted by one individual (DK).

### 2.5. Data Analysis

Due to the heterogeneity of the included studies, the results are presented using narrative synthesis, and no meta‐analysis was conducted [34]. Existing definitions of pharmacy services were adapted for use to categorise the community pharmacy depression services [35], and a new category of ‘Screening Service’ was developed. Depression service components were mapped to the Template for Intervention Description and Replication (TIDieR) checklist to describe the services (e.g. who and what the service involved and resources and materials required) [36]. The Consolidated Framework for Implementation Research (CFIR) was used to classify reported barriers and facilitators [37], whereby reported barriers/facilitators were mapped to this framework. The CFIR is a determinant implementation framework of factors influencing implementation amongst domains: innovation characteristics, the inner setting, the outer setting, characteristics of the individual, the implementation process, and implementation outcomes [38]. It has been used widely [33, 39], including within community pharmacy contexts.

### 2.6. Quality Assessment and Level of Evidence

Duplicate independent quality assessment was conducted (by DK, NW, or HA) using the Quality Assessment with Diverse Studies (QuADS) appraisal tool [40] with a third assessor acting as mediator where conflicts arose.

## 3. Results

**Figure 1:**
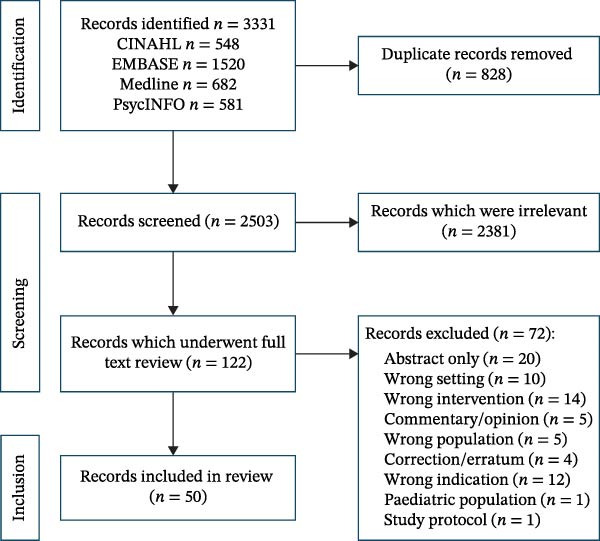
PRISMA diagram.

A total of 3331 records were identified, of which 828 were duplicates and removed. After screening, 50 publications were included (Figure 1). Two studies were translated from French and Spanish [41, 42]. The supplementary searches yielded no additional studies.

### 3.1. Characteristics of Included Studies

Of the 50 studies, 13 (26%) were conducted in the United States, 11 (22%) in Australia, five (10%) in both Canada and the United Kingdom. Other geographical regions were predominantly Europe and the Far East: four from Netherlands (8%), two from Spain (4%), and one (2%) study from each of Egypt, France, Israel, Japan, Portugal, Romania, and Thailand. Three studies (6%) did not explicitly report their geographical location. Most studies were descriptive or qualitative studies (*n* = 32, 64%), followed by randomised controlled trials (RCTs) (*n* = 13, 26%), two (4%) were case‐control or cohort studies (*n* = 2, 4%), and one (2%) was a controlled trial without randomisation. The median quality assessment score was 26/39, with a range of 8–32 (Appendix 2: Table A2). Common areas where studies were considered low in quality, as per the QuADS criteria, related to lack of underpinning theory in study design and insufficient detail regarding data collection and analysis methods.

Of the 50 studies, 33 explored interventions, whilst 17 explored general attitudes/perceptions of the concept of pharmacy‐led depression services [41, 43, 44, 50, 51, 54, 58, 62, 63, 65, 66, 72, 73, 76, 78, 81]. These studies identified general acceptance of community pharmacy–led services for people with depression [43, 58, 65, 66, 78], alongside the importance of training and resources for the pharmacy staff and addressing mental health stigma [43, 44, 51, 54, 62, 73, 76]. Six explored the concept of services focused on potential subpopulations, such as perinatal women [44, 62, 76], men [72], and the elderly population [41, 43]. In total, 21 studies reported more women than men participating [17, 18, 42, 45, 47, 49, 53, 56, 57, 61, 64, 68, 74, 75, 79, 80, 83–87]. Hereafter, due to the large number of studies identified, this review will focus on the 33 interventional studies.

### 3.2. Interventional Studies (*n* = 33)

The most common interventional studies explored depression advice/education services (*n* = 15, 30%) or screening services (*n* = 12, 24%). Services focuses on treatment adherence (*n* = 4, 8%), medication review (*n* = 1, 2%), or disease therapy management (DTM) (*n* = 1, 2%) were less commonly evaluated interventions (Table 1). Table 1 presents a description of the characteristics of the interventions, with Table 2 presenting study details and associated outcomes.

**Table 1:** Summary of the function of services explored (*n* = 33 studies) as per the (TIDieR) checklist [36].

Service	What (definition)	How/where (location)	When (initiation)	How much (frequency)
Depression advice/education service (*n* = 15 studies) [17, 18, 42, 48, 49, 55, 64, 68, 70, 71, 77, 80, 84, 87, 88]	Services for people with depression which focus on patient education about medication and/or the condition, which could involve advice, counselling, or coaching	Face‐to‐face in a private room, with option to conduct via telephone Usually delivered one‐to‐one	When patients are newly prescribed antidepressants	Involves 1–6 (most commonly 3) sessions that vary from once per week to once per 3 months, lasting 20–60 min Telephone sessions were typically shorter in duration
Screening service (*n* = 12 studies) [46, 47, 56, 57, 59–61, 67, 79, 82, 83, 85]	Application of a series of questions to identify undiagnosed depression or those at risk of depression, using validated questionnaires Participation is expanded to the wider general population	Usually face‐to‐face within the community pharmacy being administered by staff member. Usually while participants are waiting for prescriptions Occasionally conducted remotely via telephone or by handing out paper questionnaires	Participants were invited verbally or initiated the screening after seeing posters or information included in a prescription bag from the pharmacy	One or two questionnaires completed in a single session
Depression treatment adherence service (*n* = 4 studies) [52, 53, 75, 86]	Services which solely focus on improving patient adherence to antidepressants and provision of support, sometimes including delivery of cognitive behavioural therapy (CBT)	Face‐to‐face at the community pharmacy with the option to perform in a private room or via telephone	Offered to patients when newly prescribed antidepressants, when renewing, or when visiting for a refill/repeat	Usually delivered as multiple short sessions over a 2–12‐month period, with sessions conducted every 2–3 months
Medication review (*n* = 1 study) [74]	A review of patient’s medication involving patient/carer communication, which may involve people reporting adverse events (i.e. side effects) that they think are due to their medication	A medication review questionnaire is sent to patients to complete in their own time. This explored antidepressant treatment, adverse effects, and their severity	Community pharmacy staff inform applicable patients about the service when visiting the pharmacy	Participants then receive a once‐off paper survey in the mail, complete it, and then return it for review by the community pharmacy staff
Disease therapy management (*n* = 1 study) [69]	Comprehensive management of complex patients and their condition with an emphasis greater than just medicine management, for example diagnosis and prescribing. This study focused on diabetes prevention for people with depression and involved more people than just pharmacy staff (e.g. community health educators)	Typically face‐to‐face, with videoconferencing option, in numerous locations including patient’s homes and community centres		Two core sessions (1st session creates a comprehensive medication record, 2nd identified drug‐therapy problems and creates a medication action plan and report for health care providers). Up to three additional meetings offered if required

**Table 2:** Study details and associated outcomes.

Study ID, location	Description	Population (patient)	Population (pharmacist)	Study design	QuADS score	Outcomes			
						Clinical	Humanistic	Economic	Service
Depression advice/education service									
Brook et al. [55], The Netherlands	Providing education and coaching	135 participants in the trial; (intervention group: *n* = 64, control group: *n* = 71) of mixed genders and severities of depression	19 pharmacists	RCT	28	Intervention arm improved significantly more than the control on depressive symptoms and anxiety (general linear model analyses and per protocol analysis). Intention to treat with the group mean imputation method showed no differences	NR	NR	NR
Brook et al. [71], The Netherlands	Providing education and coaching	135 in trial; 107 returned drug attitude questionnaires (DAI‐30); (intervention group: *n* = 64, control group: *n* = 71) of mixed genders and severities of depression	19 pharmacists	RCT	24	NR	Intervention patients had a significantly more positive drug attitude compared to control group. Intervention patients were significantly more positive than the controls, regarding the clear explanation of ADs and helpfulness of coaching. Most would recommend drug coaching by pharmacists and positively perceived the service	NR	NR
Bosmans et al. [70], The Netherlands	Providing education and coaching	88 participants in the trial (intervention group: *n* = 40, control group: *n* = 48) of mixed genders and severities of depression	19 pharmacists, in urban and rural regions	RCT	24	[Clinical outcomes are reported elsewhere–Brook 2003]	NR	No differences in direct, indirect, or total costs. Incremental cost‐effectiveness ratio was €149 per 1% improvement in adherence and €2550 per point improvement in the depression mean item score	There were no differences in resource use between the intervention and control group
Littlewood et al. [88], England (North)	Brief psychological intervention within a collaborative care framework	Feasibility study: *n* = 24 eligible participants (of whom nine were interviewed), 51–83 years (mean 66 years), male *n* = 6, female *n* = 18, White ethnicity *n* = 24, pilot RCT: *n* = 44 eligible participants (*n* = 24 intervention, *n* = 20 usual care), 20–89 years (mean 67 years), male *n* = 21, female *n* = 23	Feasibility study: pharmacies involved *n* = 17 Pilot RCT: pharmacies involved *n* = 12	RCT	31	Slight reduction in depression symptoms at 4‐month follow‐up across both, with a slightly larger reduction in the usual‐care arm Mean episodes of depression were similar between the groups For the GAD‐7 anxiety score, at the 4‐month follow‐up in both treatment arms, this was a reduction in mean scores for both groups	There were small changes across quality‐of‐life domains in both arms, but the overall utility values reduced by the same quantity in both treatment arms	Intervention cost per participant was £51.40 (range £9.30–91), including the cost of sessions, administrative work, and clinical supervision	75% of participants allocated to the intervention took up the intervention, with 89% completing at least two sessions; the retention rate was 93.2% at the 4‐month postrandomisation follow‐up Intervention delivery (*n* = 24): received 4–6 sessions (*n* = 12, received 1–4 sessions (*n* = 9), did not commence sessions (*n* = 6)
Rickles et al. [80]; Wisconsin, USA	Telemonitoring of antidepressant use	63 participants (intervention group *n* = 28, usual care *n* = 32); 92% were white, and 84% were women; median 38 years old (range, 19–70)	14 pharmacists recruited from eight community pharmacies affiliated with a large managed health care organisation	RCT	28	Intervention did not have a significant impact on depression symptoms. Both groups showed significant reductions in symptoms from baseline to the end of the 3‐month intervention period (*p* ≤ 0.001). The percentages of patients experiencing at least a 50% improvement were not significantly different in the two groups	Intervention patients were significantly more likely to communicate with a pharmacist about their condition and treatment. The intervention arm had a significantly positive impact on antidepressant knowledge, antidepressant beliefs, and orientation toward treatment progress	NR	NR
Rickles et al. [84]; Wisconsin, USA	Telemonitoring of antidepressant use	63 participants (intervention group *n* = 28, usual care *n* = 32); 92% were white, and 84% were women; median 38 years old (range, 19–70)	14 pharmacists recruited from eight community pharmacies affiliated with a large managed health care organisation	RCT	31	NR	[As above] patient feedback was unrelated to measures of nonadherence or depression symptom after 3 months	NR	NR
Rubio‐Valera et al. [49]; Gavà and El Prat, Barcelona, Spain	Providing education/advice	179 participants (intervention group *n* = 87, usual care group *n* = 92); most were women (75%); mean age of 46.6 years; 51% met DSM‐IV criteria for major depression	Not reported	RCT	25	NR	The effectiveness analysis showed no statistically significant differences between groups in either adherence, depressive symptoms, or QoL	The effectiveness analysis showed no statistically significant differences between groups. Total costs were higher in the intervention group, mainly because of increased costs in productivity losses. Cost‐effectiveness planes showed that intervention had a probability of only 0.71 and 0.75 in terms of improvement of adherence and QoL, respectively, of being cost‐effective	NR
Rubio‐Valera et al. [68]; Gavà and El Prat, Barcelona, Spain	Providing education	179 total, randomised to the CPI (*n* = 87) or UC group (*n* = 92) Most participants were women (75%), with mean age of 46.6 years. 51% of the participants met DSM‐IV criteria for major depression	Not reported	RCT	27	Both groups showed an improvement in symptoms at 3 and 6 months. No differences in symptom severity were observed between groups	Intervention group more likely to remain adherent at 3 and 6 months but not statistically significant. NNT was 5. A significant time group interaction was found in health‐related quality of life tariffs in favour of the intervention group. Overall improvement was higher in the intervention group	NR	NR
Rubio‐Valera et al. [42]; Gavà and El Prat, Barcelona, Spain	Providing education	179 participants; 113 whose symptoms had remitted at 6 months selected for secondary analysis (intervention group [GI] = 58; control group [CG] = 55); mostly women (64%–82%)		RCT	18	The proportion of relapses was lower in the intervention group at 18 months after starting treatment but was not statistically significant	NR	NR	NR
Phimarn et al. [48]; Thailand (North‐eastern)	Group and individual counselling	68 students with experience of depression (nursing, medicine, pharmacy, and public health) Group counselling by pharmacist (*n* = 34), male *n* = 22, female *n* = 12; mean age 20 years old; individual counselling by pharmacist (*n* = 34), male *n* = 22, female *n* = 12; mean age 19.9 years old	1 university pharmacy	RCT and descriptive/qualitative study	30	Depression scale (CES‐D) scores of both groups significantly reduced from the baseline. The decreased score was greater when counselled individually	At week 16, counselling significantly increased QoL score of physical health, whereas the mean score of mental health was increased significantly only by individual counselling. Both counselling types significantly increased mean score of physical health, whereas score of mental health was increased significantly only by the individual counselling	NR	NR
Prats and Cebrián [87]; location not reported	Providing educating and monitoring	13 people diagnosed with depression; women *n* = 11, men *n* = 2; age range 21–67 years (average 42.7); 12 diagnosed with treatments for >1 year	NR	Descriptive/qualitative study	25	NR	Drug‐related problems detected included: misdiagnosis of depression, patients inappropriately stopping medication, nonadherence, and side effects	NR	NR
Crockett et al. [64]; New South Wales, Australia	Providing education and monitoring	106 participants in trial (intervention group: *n* = 46, control group: *n* = 60) mean age 46 years, predominantly female and not currently employed	32 community pharmacies, rural and remote	Quasiexperimental (cluster randomised)	22	NR	No difference in adherence between control and trial participants, and no change in drug attitude Well‐being scores decreased significantly in both groups	NR	NR
Chew‐Graham et al. [17]; England (North East)	Brief psychological intervention within a collaborative care framework	24 participants; 51–83 years (mean 66 years); male *n* = 6, female *n* = 18; White ethnicity *n* = 24; hypertension (*n* = 16). [*n* = 11 patients participated in interview]	8 community pharmacies in rural and urban settings in the northeast of England. [*n* = 9 facilitators participated in interview; *n* = 5 facilitators participated in a focus group]	Descriptive/qualitative study	26	Reported elsewhere–Littlewood 2002	NR	NR	Average recruitment rate of 0.55 participants per community pharmacy per month
Gorton et al. [18]; Greater Manchester, UK	Providing education and monitoring	76 participants; median age 39 (IQR 28–47); 62% women; sertraline was the most commonly prescribed medication (46%)	10 pharmacists in 9 pharmacies had begun recruiting patients at the point of their initial interviews 4 pharmacists were able to participate in the follow‐up interviews	Descriptive/qualitative study	21	NR	Patients described a range of major concerns such as mental and physical health generally, trauma, bereavement and work. Few (*n* = 7) highlighted medication as a priority Pharmacists documented consultation focused on life experience (*n* = 51), medication (*n* = 47), health (*n* = 42), support solutions (*n* = 36), and patients’ expression of their feeling (*n* = 31)	NR	NR
Shiamptanis et al. [77]; Sudbury and Espanola, Ontario, Canada	Providing education and follow‐up	30 participants; average of 2 follow‐ups	5 providers (3 physicians and 2 pharmacists) 18 total (7 physicians and 11 pharmacists)	Descriptive/qualitative study	29	Pharmacists identified drug‐related problems in 13 (43.3%) patients and provided pharmaceutical opinion/intervention to 10 (33.3%) patients. 12 (40.0%) received additional support on healthy sleep habits, 8 (26.6%) on physical activity, 5 (16.6%) on nutrition, and 1 (3.3%) on yoga	NR	NR	NR
Screening services									
Ballou et al. [59]; Wilkes County, North Carolina, USA	Providing depression screening	77 surveys completed across three methods (bag stuffer, personal ask, and interview); mostly female; median age 51.5; 31% previous or present diagnosis of depression, 22% currently or historically treated for depression	1 independent community pharmacy	Descriptive/qualitative study	27	Most participants scored <10 on PHQ‐9, indicating no or mild depression; 18/77 participants required referral for treatment; 4/77 required activation of the emergency protocol at level 1 or 2; 0/77 required a level three measure of care	93% (72/77) rated depression screening service at least somewhat valuable; 53/77 participants rated ‘very’ or ‘extremely’ valuable; all 18 that scored 10 or higher on the PHQ‐9 rated either ‘very’ or ‘extremely’ valuable	NR	1/50 (2%) questionnaires were returned in the bag stuffer group 36/50 (72%) questionnaires were returned in the personal ask group 40/50 (80%) questionnaires were returned in the interview group
Condinho et al. [85]; Portugal	Providing depression screening	591 participants, (*n* = 441; 74.9% females). Mean age was 50.2 years (SD = 16.6)	28 pharmacies invited according to their geographical distribution in terms of accessibility to the research team	Descriptive/qualitative study	12	GAD‐7: amongst people without a diagnosis of anxiety (*n* = 477), 18.2% had moderate to severe symptoms (score ≥10). Of those diagnosed with anxiety (*n* = 94), 39.4% had moderate to severe symptoms, indicating a lack of control over the condition PHQ‐9: Amongst those without a diagnosis of depression (*n* = 485), 12.1% also had moderate to severe symptoms (score ≥10). Amongst those diagnosed with depression (*n* = 97), 49.5% also had moderate to severe symptoms	NR	NR	NR
Gide et al. [60]; New South Wales, Australia	Providing depression screening	15 patients aged 65+ with current diagnosis of chronic illness, experienced a medical or personal event in the past 12 months, taking 5+ regular medications, experiencing side effects, and/or risk of social isolation (67% female) Five patients participated in semistructured interviews	8 community pharmacies performed screening (39 community pharmacies received training) 6 pharmacists participated in semistructured interviews	Descriptive/qualitative study	27	67% received a GDS‐15 score of 6+, indicating possible depression and requiring referral. Pharmacists referred 80% of patients to another HCP. One patient was diagnosed with depression and commenced antidepressant therapy	Pharmacists and patients viewed these screening services as acceptable and useful, with pharmacists reporting an increase in comfort and confidence regarding screening	NR	Pharmacists were found to be capable of delivering late‐life depression screening services following training
Habba et al. [83] Michigan, USA	Providing depression screening	70 participants aged 18–90 (mean 52), 53 female, 65 non‐Hispanic	25 student pharmacists and 10 community pharmacists and faculty preceptors attended the training sessions	Descriptive/qualitative study	21	90%+ of participants completed the 2‐week follow‐up, and 92.3% reported being comfortable seeking mental health services from a pharmacist 53.8% reported reading the educational materials, 24.6% helped a friend or family member, and 16.9% made an appointment with their health care provider	PHQ‐9 scores in participants younger than 55 years of age were nearly 2 times higher than those older		Innovative student pharmacist–led model increased access to depression screenings, education, and referral services within a community pharmacy setting
Hare and Kraenow [79]; Kansas City metropolitan area, USA	Providing depression screening	18 participants; 16 women, 2 men; 16 white, 1 black, 1 Asian; mean age 56	6 community pharmacists and 4 psychiatric pharmacy specialists	Descriptive/qualitative study	19	78% (*n* = 14) were unlikely to have major depressive disorder; 17% (*n* = 13) had symptoms consistent with current MDD; 6% (*n* = 1) had symptoms strongly consistent with depression; recommendations made to 6 patients screened (33%): immediately transported to the hospital (*n* = 1), recommended continuation of medication/counselling (*n* = 2); referral (*n* = 3)	82% of the patients screened were satisfied (*n* = 3) or very satisfied (*n* = 11) with the service; none was dissatisfied/very dissatisfied All said that they would participate in future screenings in the pharmacy setting	NR	NR
Jewell et al. [67]; Rural Southeast Ohio, USA	Providing depression screening	61 patients identified by insurance as taking a mental health medication for anxiety (*n* = 24) and depression (*n* = 37)	4 pharmacies in a multistore, independent pharmacy	Descriptive/qualitative study	26	Mean GAD‐7 scores were 8.1 at initial appointment and 6.4 at follow‐up. This showed a relative 20% reduction in GAD‐7 scores Mean PHQ‐9 scores were 11.2 at initial appointment and 10.0 at follow‐up. This showed a relative 10% reduction in PHQ‐9 scores	Borderline significant change in scoring shows the possibility of improving patient mental well‐being with pharmacists supporting providers and patients in plan implementation and follow‐through	NR	Results highlighted the possibility of implementing a mental health service to address medication adherence and lifestyle changes and support provider visits in a rural community May offer a potential way to increase mental health screening in accordance with the guidance from the U.S. Preventive Services Task Force
Knox et al. [56]; California, USA	Providing depression screening	25 participants; 16 women, 9 men; 18 students, 5 faculty/staff, 1 alumnus, 1 parent of a student; age range 18–53 years, majority between 18 and 22 years of age	NA	Descriptive/qualitative study	32	Mean score on the Zung Self‐Rating Depression Scale was 32.04; only one participant scoring greater than 50. Two formal referrals made as one participant scored 48, placing that individual in the ‘borderline depression’ range	Most participants reported that the survey was very useful, they were very comfortable completing the survey, they were very or somewhat likely to read the provided information, and they had learned something about depression or themselves by participating in the survey	NR	NR
Kondova et al. [46]; Bulgaria	Providing depression screening	83 participants mean age 57.8 years	NA	Descriptive/qualitative study	29	70% were positive for depressive symptoms; 55% of the individuals had indications of mild to moderate depression and were directed to a psychiatrist	NR	NR	NR
O’Reilly et al. [47]; New South Wales, Australia	Providing depression screening	41 participants; mostly female (*n* = 29); most ages 18–40 years	20 pharmacists from 12 participating pharmacies	Descriptive/qualitative study	29	70.7% of participants were referred to a healthcare provider	PHQ‐9 was the most commonly used tool (*n* = 22) considered more comprehensive. WHO‐5 was 2nd most used (*n* = 15) with pharmacists commenting that the tool was preferable due to the positive wording of the items, making it less confronting to use as a screening tool. Beyondblue Depression Checklist was the least used (*n* = 8), some pharmacists found it a helpful tool to use as many of their patients felt at ease with it as they were familiar with the Beyondblue organisation	NR	NR
Reid et al. [57]; USA (Midwest)	Providing depression screening	41 participants aged 18–45 (female = 27) fluent in English with specific mental health diagnosis (anxiety (28), depression (26), PTSD (4), Schizophrenia (1), bipolar disorder (1))	7–8 pharmacists, ~6 technicians, 1 postgraduate (year 1) community pharmacy resident	Descriptive/qualitative study	28	29 patients screened for anxiety and depression; of these, 41% scored in the high‐risk category for depression or anxiety and met with the pharmacist for the consultation Pharmacist identified 1–2 medication‐related problem for each participant, often related to undertreatment. The most common was the need for additional therapy and inadequate dosages			Most patients flagged for the depression screening did not discuss the screening offer with the resident For patients offered the screening by the resident pharmacist, most (70.7%) were willing to complete the depression and anxiety screening
Rosser et al. [61]; Ohio, USA	Providing depression Screening	3726 participants; 53% women, mean age 53 years	NA	Descriptive/qualitative study	27	PHQ‐2: 67 (1.8%) screened positive; PHQ‐9: a total of 17 (25.3%) would warrant consideration of diagnosis or modification of treatment. All patients with a positive PHQ‐9 were referred to a physician	NR	NR	NR
Zmoira et al. [82]; USA	Providing depression screening	69 participants	NA	Descriptive/qualitative study	28	12 (17.4%) screened positive on the PHQ‐2, and 6 patients (8.7%) screened positive on the PHQ‐9. These patients were referred to their provider to consider a depression diagnosis or monitoring depression symptoms	NR	NR	NR
Depression treatment adherence service									
Brook et al. [75]; The Netherlands	Providing education and coaching to improve adherence	135 participants (Intervention group *n* = 64, control group *n* = 71); mean ± SD age was 43 ± 13 years; mostly women (70%); most had moderate to severe symptoms (82%)	19 community pharmacists in various regions of the Netherlands–(same as Brook 2003)	RCT	23	NR	Intention‐to‐treat analyses showed no intervention effect on adherence, whereas analyses of patients who received the intervention showed improved adherence (73% compared with 90%). Neither analysis showed effects on depressive symptoms	NR	NR
Klang et al. [53]; Israel	Providing advice and reminders	173 participants in intervention group; 49 men (28%) 124 women (72%) mean age 53.9 ± 18.9 years (range: 21–91) 12,746 patients experienced ‘treatment‐as‐usual’ (TAU) (i.e. prescribed escitalopram by their family physician in the same 17 centres where the study was carried out)	17 pharmacies	Cohort study	31	NR	At 1 month, the adherence rate was 71% in the pharmacist arm and at 6 months, the rates were 55% versus 1‐month adherence rates of 57% in the TAU (treatment as usual) arm; at 6 months, the rate was 15.2% (*N* = 1934) in the TAU arm (compared with CP rates: *p* < 0.0001)	NR	NR
Shoji et al. [52]; Osaka and Hyogo Prefectures, Japan	Cognitive behavioural therapy	31 participants (intervention group = 19, control = 12) diagnosed with unipolar depression, currently receiving antidepressant prescriptions, and visiting the participating pharmacy for prescription refills	4 pharmacies (intervention = 2, control = 2) that accept prescriptions mainly from psychosomatic medicine and psychiatry and routinely provide medication to depressive patients	Quasiexperimental (nonrandomised trial)	30	Intervention: mean DAI‐10 score increased from 4.941 at baseline to 6.105, the mean PHQ‐9 score decreased from 9.263 to 8.625, and the mean patient satisfaction score increased from 39.947 to 42.211 Control: mean DAI‐10 score decreased from 6.333 to 4.167, the mean PHQ‐9 score increased from 9.333 to 12.923, and the mean patient satisfaction score decreased from 38.929 to 38.167	Intervention‐group patients showed improvements in clinical symptoms	NR	NR
Bultman and Svarstad [86]; Wisconsin, USA	Patient interview and monitoring	100 participants; mean age 36; mostly women (76%) and white (89%)	23 community pharmacies	Descriptive/qualitative study	8	NR	Adherence: 24% reported stopping the antidepressant within 2–3 months; 83% reported nonadherence during study period Knowledge of medication: 81% knew about adverse effects; 76% were aware of means of coping with adverse effect; 64% knew antidepressants require time before experiencing benefits; 30% knew recommended treatment; 40% could explain how antidepressant worked Perceptions: 93% viewed antidepressant as a good option; 30% agreed adverse effects would be bothersome; 7% indicated they would prefer a different medication; taking daily was viewed as not harmful by 86% Satisfaction: 57% reported feeling better a lot of the time, and 30% some of the time; 75% reported antidepressant did not bother them/a little; 47% very satisfied with antidepressant	NR	Monitoring: 17% of patients had no further contact with a pharmacist concerning their antidepressant medication; 75% were asked whether they had any questions; 53% felt the pharmacist encouraged them to ask questions; 54% reported that the pharmacist listened to their concerns; pharmacists were reported to solve medication problems (32%)
Medication review service									
Farcas et al. [74]; Cluj‐Napoca, Romania	Medication review for adverse reactions	50 participants (participated in questionnaire); mostly female (68%); average age 45.8 years (range 20–82)	12 community pharmacies in Cluj‐Napoca	Descriptive/qualitative study	31	All reported an adverse event; total number reported was 375; median per patient was 7.5 (range 1–26); 54% percent of the total number of the reported adverse events was classified as being probably caused by the antidepressant, with 45% classified as being possible	NR	NR	NR
Disease therapy management									
Wagner et al. [69]; Connecticut, Massachusetts, or Rhode Island, USA	Intervention to decrease risk of diabetes in people with depression	188 participants Control (*n* = 53) Intervention (*n* = 70) Intervention + DTM (*n* = 65)	NA	RCT	27	NR	NR	NR	Treatment fidelity was high; participants reported treatment satisfaction, group cohesion, and therapeutic alliance to pharmacists.

Abbreviations: CES‐D, Centre for Epidemiologic Studies Depression Scale; DAI‐10, drug attitude inventory‐10; DAI‐30, drug attitude inventory long version; DTM, disease therapy management; GAD‐7, general anxiety disorder‐7; IQR, interquartile range; NR, not reported; PHQ‐2, patient health questionnaire‐2; PHQ‐9, patient health questionnaire‐9; QoL, quality of life.

#### 3.2.1. Depression Advice/Education Service Outcomes (*n* = 15)

Clinical outcomes for depression advice/education services related to symptoms of depression (*n* = 5), anxiety (*n* = 2), or drug‐related problems (*n* = 2). Of the studies that explored details of depression symptoms, three identified improved symptoms of depression when compared to those who did not receive any education. One study reported that the majority of participants achieved remission at 6 months [55], whilst others reported slight reduction in symptoms [48, 88]. Two studies reported that people who experienced the service had improvements in depression symptoms; however, there was no discernible difference when compared with people who received usual care [68, 80]. There was evidence of reduced anxiety when compared with those who did not receive the service [55, 88]. One study [87] identified up to nine drug‐related problems that would otherwise have gone unnoticed, such as side effects, whilst another identified drug‐related problems in 43% of participants and provided necessary support [77].

Humanistic outcomes included medication adherence, quality of life (QoL), and perceptions of the services. Of the four studies that reported medication adherence, three [64, 84, 87] found no improvement in adherence with antidepressants, with another study reporting nonadherence related to wider drug‐related problems [68]. Four studies [18, 48, 64, 88] identified improvements in QoL, one of which [48] identified a significant increase when compared to usual care. Two studies [71, 80] identified an impact on public perception with participants reporting more positive perceptions about their progress or they would recommend the services to others. Regarding attitudes to medication, one study [71] identified significantly more positive attitudes from participants towards antidepressant medication, though another found no significant changes [64]. One study [80] reported people who experienced the service had significantly better knowledge of antidepressants than those who had not, and that patients were more likely to communicate with the pharmacist because of the service.

Relating to cost‐effectiveness, two studies [49, 70] concluded that depression advice/education services were unlikely to be cost‐effective in terms of improving remission symptoms, adherence, or quality‐adjusted life‐years. The study based in the United Kingdom [88] identified the cost per participant was £51.40 (range £9.30–£91.00), including the cost of sessions, administrative work, and clinical supervision. One study identified no significant differences in health care resource use by people (e.g. visits to healthcare professionals) for those who experienced the service [70].

#### 3.2.2. Screening Service Outcomes (*n* = 12)

All 12 screening service studies [46, 47, 56, 57, 59–61, 67, 79, 82, 83, 85] reported clinical outcomes relating to the identification of depressive symptoms, with the majority of general population patients scoring low (no‐mild depressive symptoms) with the screening tools used. Three studies [56, 60, 79] reported the proportion of patients requiring further referral for depression services after screening, which was typically 25% or less (range = 8%–55%), with one study [60] showing that 80% of those screened were referred to another HCP. Four screening services reported positive humanistic outcomes related to the perceived value of the service to patients [56, 59, 60, 79]. The first [59] reported that 93% of participants thought the service was valuable, with 100% of people identified as at risk of depression during the screening service considering it either very or extremely valuable. Another [56] found high levels of satisfaction with all participants describing the service as very (64%) or somewhat (36%) useful and that the experience was very (96%) or somewhat comfortable (4%). The third [79] reported patients being either satisfied (17%) or very satisfied (61%) with the service. The final study [60] noted both pharmacists and patients viewing screening services as acceptable and useful, with pharmacists reporting an increase in comfort and confidence regarding screening.

#### 3.2.3. Depression Treatment Adherence Service Outcomes (*n* = 4)

Three of these services noted multiple humanistic outcomes, reporting the impact the service had on patients’ medication adherence, with two studies reporting significant increases in adherence for participants receiving the service when compared to treatment as usual (TAU). This was 90% vs. 73% in one study [75] and 71% vs. 57% at 1 month, then 55% vs. 15.2% at 6 months in the other [53]. A third study [86] identified 24% of patients stopped their antidepressant within 2–3 months, and 83% of people had either forgotten to take their antidepressant, chose not to take it, or took extra medication. This study also identified the adherence service to have a positive impact on peoples’ knowledge, their attitudes to medications, and their ability to ask the pharmacist questions. The final study [52] noted that the intervention group who received cognitive behavioural therapy (CBT) showed improvements in clinical symptoms but reported no other humanistic outcomes.

#### 3.2.4. Other Services (*n* = 2)

For the study that explored the medication review services, participants [80] reported at least one adverse event associated with their antidepressant, with over half (54%) reporting more than five adverse events. The most common adverse events included fatigue, somnolence, excessive sweating, weight gain, and sexual disorders. Of these reported adverse events, 99% were classified as being probably (54%) or possibly (45%) caused by the antidepressant. For the drug therapy management intervention [69], participants reported good interaction and relationship with the health care professionals involved; 98% of participants attended at least one session with the community pharmacist, and 77% of people completed ≥4 sessions.

### 3.3. Implementation Barriers/Facilitators

Several barriers and facilitators were identified from 20 studies (Table 3) [17, 18, 42, 46, 47, 52, 56, 57, 60, 67–69, 77, 79, 80, 82–84, 86, 88]. When aligning to the CFIR framework, the most common barriers reported related to the ‘outer setting’; with local attitudes (e.g. stigma and lack of awareness of pharmacy setting), financing/funding restraints, and limited collaboration with other healthcare professionals being reported across studies. In terms of ‘innovation design’—that is the community pharmacy depression services—there were no reported barriers in this domain. Many facilitators related to the ‘inner setting’ in terms of the accessibility of pharmacies, the availability of a private consultation room, and provision of training and resources supporting the implementation of the services. Conversely, a prominent barrier also related to the ‘inner setting’ in relation to time and burdensome implementation. The perceived acceptability of the service and it being positively perceived by patients, as well as the appropriateness of the community pharmacy setting, were identified as facilitators.

**Table 3:** Reported barriers, facilitators, and anticipated facilitators (reported by *n* = 20 studies).

CFIR domains Subdomains	Barriers	Facilitators	Anticipated facilitators
Innovation design			
Innovation adaptability		Preferred face‐to‐face option (*n* = 1) [18]	
Innovation design		Quick to conduct (*n* = 3) [46, 47, 60]	
Outer setting			
Local attitudes	Stigma (*n* = 5) [46, 57, 60, 79, 82] Low awareness of community pharmacy staff role (*n* = 5) [47, 57, 60, 82, 86] Patient reluctance to seek help (*n* = 3) [57, 60, 86] Familiarity with staff (*n* = 2) [79, 88]	Overcoming stigma/misinformation (*n* = 5) [18, 77, 80, 82, 88]	Greater patient relationships (*n* = 6) [18, 52, 57, 60, 84, 88]
Partnership and connections	Lack of collaboration with other healthcare professionals (*n* = 4) [57, 67, 82, 84]	Collaboration with other healthcare professionals (*n* = 3) [42, 56, 77]	Greater relationship with other healthcare professionals (*n* = 4) [17, 42, 47, 77]
Financing	Financial restraints/lack of cost‐effectiveness (*n* = 6) [17, 47, 68, 80, 82, 88]		
Inner setting			
Available resources	Lack of time/burdensome implementation (*n* = 6) [17, 56, 57, 60, 82, 88]	Monitoring tools for pharmacists (*n* = 2) [67, 86]	
Structural characteristics (physical infrastructure)		Accessibility of community pharmacies (*n* = 6) [17, 18, 46, 56, 60, 82] Confidentiality/private space (*n* = 4) [17, 46, 47, 82]	
Access to knowledge and information		Training, tools, and study material (*n* = 5) [17, 56, 60, 67, 88]	Improved training/transferrable skills (*n* = 6) [17, 47, 56, 77, 83, 86]
Individuals			
Characteristics	Staff lacking confidence/emotional strains (*n* = 6) [17, 18, 47, 82, 83, 86]		Staff motivation (*n* = 1) [82]
Implementation			
Engaging	Challenges recruiting/lack of patient involvement (*n* = 11) [17, 18, 42, 46, 57, 68, 69, 79, 82, 83, 88]	Displays that raise awareness (*n* = 2) [47, 82]	
Implementation outcomes			
Acceptability		Patient satisfaction (*n* = 7) [17, 52, 57, 69, 77, 80, 83]	
Appropriateness		Appropriate setting (*n* = 5) [17, 46, 47, 60, 88]	

## 4. Discussion

This systematic review identified a range of service types to support people with depression from community pharmacies across various different geographic locations. The variety of services reflects the complexity of depression and the multiple ways in which patients could be supported by pharmacy staff. There were generally positive perceptions towards community pharmacy–led depression services. Services offering advice or education about the condition or its treatment were the most common, and a range of barriers and facilitators were identified mostly within the pharmacy ‘inner setting’ and the external ‘outer setting’.

Despite the large number of studies on community pharmacy depression services (*n* = 50), few presented strong empirical evidence on the beneficial clinical, humanistic, and economic outcomes of community pharmacy depression services. Most studies were descriptive/qualitative in nature, rather than clinical trials, which impacted the identification of comparative benefits [89]. A Cochrane review of pharmacists’ services for nonhospitalised patients in 2018 also identified limited evidence of pharmacists’ influence on depression‐related outcomes [90]. It is therefore difficult to determine which services would be most beneficial in supporting people with depression. Considering the studies in this review were small‐scale trials/pilots, to support wider spread implementation at regional/national levels, future research is needed to investigate service outcomes in higher‐quality formats such as clinical trials. In the absence of such research, consensus methodology may be the most appropriate approach to identify local priorities and direct future service development.

Most services focused on offering advice and education about depression and/or its treatment, or screened people for their risk of depression. This is perhaps unsurprising given the long‐standing tradition of community pharmacy in educating the public and offering screening services for various clinical conditions [91–94]. However, the education/advice services identified in this review represents a shift to offering a more in‐depth/bespoke educational session, moving beyond general drug‐related counselling (e.g. on side effects). Such services are delivered by community pharmacy staff to the public for a range of other conditions [95], including osteoporosis [96] and cancer [97], representing the expanded scope of practice of community pharmacy [98]. In the United Kingdom, there is a drive to position community pharmacy as information hubs and to be more directly involved in clinical care. For example, ‘Pharmacy First’ initiatives across the UK represent funding models whereby pharmacies are reimbursed for offering advice, treatment, and onward referral [99], with community pharmacies seen as the ‘first port of call’ for patients rather than a primary care physician [100]. Additionally, the extent of community pharmacy depression services identified on education, advice, and screening perhaps challenges previous perspectives that community pharmacists are uncomfortable offering care to people with mental health conditions and have limited time for this patient population [101]. On the contrary, this review provides evidence that community pharmacy is well placed to offer accessible primary care mental health support [102].

Services offering in‐depth medication reviews and drug therapy management associated with depression and its risks were limited within community pharmacy, despite the fact that numerous pharmacist‐led clinical medication reviews have been trialled in community pharmacy for various other conditions such as asthma, cardiovascular disease, and diabetes [103]. It is plausible that the limited involvement of community pharmacy staff with medication reviews involving antidepressants relates to two key barriers/facilitators identified from this review. Firstly, the lack of collaboration which can exist between community pharmacy staff and local mental health physicians may hinder pharmacies’ involvement in medication reviews/drug therapy management for depression, as previous research suggests pharmacists’ involvement with psychiatric care strongly benefits from a multidisciplinary and collaborative approach [24, 104]. Secondly, the importance of sufficient education and training to enable pharmacy staff to offer depression care was a prominent finding of this review and others [102]. It is likely that in‐depth medication reviews and drug therapy management may require greater training and support, as it is acknowledged that pharmacists with limited specialist skills may not be sufficiently trained to approach complex clinical cases and may lack sufficient knowledge of specialised use of mediation for mental health conditions [105, 106].

Analysis of the barriers/facilitators identified interesting insights, as they mostly related to the ‘inner’ and ‘outer’ setting which aligns with previously known literature on factors impacting implementation success of community pharmacy services [107]. This includes the importance of engaged pharmacy staff who are knowledgeable with sufficient competency and capability to deliver services, as well as external engagement including collaboration with other healthcare professionals and the positive local attitudes of patients [107, 108]. However, some unique differences in relation to commonly cited barriers/facilitators were identified when comparing to the broader literature [107]. Firstly, the innovation design of the depression services themselves was not cited as a barrier, with no challenges related to complexity and operationalisation of the service. This is usually a commonly reported barrier with community pharmacy innovations, and our divergent findings suggest the feasibility and utility of depression services within community pharmacy practice [107]. Secondly, although the importance of the accessible location of community pharmacies has been well documented as a key facilitator for patients [108], structural characteristics associated with the importance of a private consulting space were more commonly cited in this review. This may reflect broader societal needs for accessible, yet discrete and confidential, mental healthcare support—which are known quality indicators for care of depression within primary care [109]. A focus on implementation facilitators will be crucial for larger‐scale implementation of community pharmacy depression services, considering all studies in this review were short‐term pilot/trials, with none representing ‘care as usual’. Effort should be made to align barriers and facilitators to tailored and optimised implementation strategies, for example using the Expert Recommendations for Implementing Change (ERIC) taxonomy [110]. It was an interesting observation that many studies reported more women participating than men. The reason for this is unclear, although it could relate to the demographics of people who take antidepressants and/or who are more likely to seek/accept help from a community pharmacy service. Future implementation strategies should also consider how to make the services acceptable and accessible to all.

### 4.1. Strengths and Limitations

A strength of this systematic review was the international perspective. Multiple databases were accessed to collect a wide reach of studies with non‐English papers translated for inclusion. The use of Google Translate may have resulted in errors in translation, but generally, this software is found to have a high level of accuracy [111]. All papers underwent quality assessment to give indication on the strength of the evidence base. Extracted data were mapped to the TIDieR checklist [36] and the CFIR framework [37], which facilitated comparison to broader implementation science literature. The heterogeneity of the study designs meant it was not possible to conduct any meta‐analysis or weight the identified barriers and facilitators and deduce relative influences. Not all stages were conducted independently and in duplicate, which increases the risk of researcher bias. However, the use of a data extraction template which was piloted mitigated this.

## 5. Conclusion

The review identified a heterogenous array of international community pharmacy depression services, with limited evidence on empirical outcomes. This will likely act as a barrier to larger‐scale implementation of such services, and we recommend larger‐scale, high‐quality RCTs to further build the evidence base. Depression services focusing on screening and offering education may be especially suited within community pharmacies and align with broader trends positioning pharmacies as accessible health information hubs. In general, community pharmacies were identified as an appropriate location for providing depression care, considering the accessibility of their location, albeit key barriers were identified in this review which will need to be addressed for scale‐up efforts.

## Funding

This work was funded by a grant from Pharmacy Research UK (Funder Project Reference: RGS\R2\222202).

## Conflicts of Interest

The authors declare no conflicts of interest.

## Data Availability

Any additional data extraction is available upon request.

## Appendix 1

**Table A1:** Search strategy.

Search terms	Medline search^a^	EMBASE search^a^	PsycINFO search	CINAHL search
Search terms for pharmacy:	Community pharmacy services/pharmacies/pharmacists/community pharmac^b^ Retail pharmac^b^	Community pharmacist/“pharmacy (shop)”/Community pharmac^b^ Retail pharmac^b^	Community pharmac^b^ Retail pharmac^b^	Community pharmac^b^ Retail pharmac^b^
Search terms for depression services:	Depression/exp depressive disorder/community mental health services/mental health/exp antidepressive agents/depress^b^ Antidepressant^b^	Depression/mental health/community mental health/mental health service/antidepressant agent/depress^b^ Antidepressant^b^	Depress^b^ Mental adj health Antidepressant^b^	Depress^b^ Mental adj health Antidepressant^b^

^a^Associated subheadings were included using the ‘explode’ funding in OVID databases for Medline and EMBASE.

^b^Truncation code.

## Appendix 2

**Table A2:** Quality assessment of included studies using the QuADS tool.

Study ID	QuADS areas													Total score
	1	2	3	4	5	6	7	8	9	10	11	12	13	
Gide et al. [43]	1	3	3	3	3	3	3	3	3	2	3	2	1	33
Pham [44]	2	2	2	3	2	3	3	3	3	2	3	2	3	33
Littlewood [45]	3	3	3	3	3	0	3	3	3	0	3	3	2	32
Gorton et al. [18]	1	3	3	3	3	3	3	3	3	0	3	0	3	31
Kondova et al. [46]	2	3	3	3	3	2	3	1	3	0	3	2	3	31
O’Reilly et al. [47]	1	3	3	3	3	3	3	3	3	0	3	0	3	31
Phimarn et al. [48]	2	3	3	3	3	2	3	3	3	0	3	0	3	31
Rubio‐Valera et al. [49]	2	3	3	3	3	3	3	3	3	0	3	0	2	31
Gide et al. [50]	1	2	3	3	3	3	3	2	1	3	3	1	2	30
Guillaumie et al. [51]	1	3	3	3	3	3	3	3	3	0	3	0	2	30
Shoji et al. [52]	2	3	2	3	2	3	3	1	2	3	3	0	3	30
Klang et al. [53]	2	3	3	3	3	0	3	1	3	0	2	3	3	29
Mospan et al. [54]	1	3	3	3	3	2	3	3	3	0	3	0	2	29
Brook et al. [55]	1	2	2	3	3	1	3	3	3	3	3	0	2	28
Knox et al. [56]	2	3	3	3	3	0	3	3	3	0	3	0	2	28
Pham et al. [44]	2	3	3	3	3	1	3	3	1	0	3	1	2	28
Reid et al. [57]	1	2	3	3	3	2	3	3	3	0	3	0	2	28
Rubio‐Valera et al. [42]	1	3	3	3	3	2	3	3	1	0	3	0	3	28
Soliman [58]	3	3	3	3	3	0	3	3	2	0	2	0	3	28
Ballou et al. [59]	1	3	3	3	3	2	3	3	2	0	3	0	2	27
Gide et al. [60]	1	3	3	3	3	2	2	2	2	1	3	0	2	27
Rosser et al. [61]	1	3	3	3	3	0	3	3	3	0	3	0	2	27
Silverio et al. [62]	2	3	3	3	3	1	3	3	1	0	3	0	2	27
Strowel et al. [63]	1	3	1	3	3	2	3	1	2	2	3	1	2	27
Crockett et al. [64]	1	2	3	3	3	0	3	3	2	0	3	0	3	26
Gardner et al. [65]	1	3	2	1	3	0	2	3	2	0	3	3	3	26
Guillaumie et al. [66]	1	3	3	3	3	0	3	2	3	2	3	0	0	26
Jewell et al. [67]	1	3	3	3	3	2	3	1	2	0	3	0	2	26
Chew‐Graham et al. [17]	2	2	3	3	2	0	2	2	3	2	3	0	1	25
Rubio‐Valera et al. [68]	1	3	3	3	1	0	2	3	1	3	3	0	2	25
Wagner et al. [69]	1	2	3	3	3	3	3	0	3	2	0	0	2	25
Acheuk et al. [41]	1	2	2	3	2	1	2	3	1	0	3	2	2	24
Bosmans et al. [70]	1	3	3	3	3	0	2	1	3	0	3	0	3	24
Brook et al. [71]	1	3	2	2	2	1	2	3	3	0	3	0	2	24
Brydges et al. [72]	2	2	2	3	2	0	2	3	2	0	3	1	2	24
Crockett and Taylor [73]	1	3	2	3	3	0	3	2	3	0	3	0	1	24
Farcas et al. [74]	2	3	3	1	1	0	3	3	3	0	3	0	2	24
Brook et al. [75]	0	2	2	3	3	0	3	3	2	0	3	0	2	23
Elkhodr et al. [76]	1	2	3	3	1	1	3	3	2	0	3	1	0	23
Shiamptanis et al. [77]	1	3	3	3	0	1	3	1	3	0	3	0	2	23
Guillaumie et al. [78]	1	2	2	3	3	0	1	0	3	2	3	0	2	22
Hare and Kraenow [79]	1	3	1	3	1	1	3	3	1	0	3	0	2	22
Rickles et al. [80]	1	3	3	1	3	1	1	2	3	0	1	0	3	22
Stewart et al. [81]	1	1	3	2	3	2	1	3	2	1	2	0	1	22
Zmoira et al. [82]	1	3	3	3	1	3	2	1	2	0	1	0	2	22
Habba et al. [83]	1	1	3	2	3	2	1	3	1	0	1	0	3	21
Rickles et al. [84]	1	3	3	1	3	0	1	3	1	0	1	0	3	20
Condinho et al. [85]	1	1	1	1	1	0	1	1	1	2	1	1	0	12
Bultman and Svarstad [86]	1	2	1	0	0	1	1	2	0	0	0	0	2	9
Prats and Cebrián [87]	1	2	1	1	0	1	1	1	0	0	1	0	0	8

*Note:* 0 = no mention at all; 3 = full, explicit, and detailed mention. 1, theoretical or conceptual underpinning to the research; 2, statement of research aim/s; 3, clear description of research setting and target population; 4, the study design is appropriate to address the stated research aim/s; 5, appropriate sampling to address the research aim/s; 6, rationale for choice of data collection tool/s; 7, the format and content of data collection tool are appropriate to address the stated research aim/s; 8, description of data collection procedure; 9, recruitment data provided; 10, justification for analytic method selected; 11, the method of analysis was appropriate to answer the research aim/s; 12, evidence that the research stakeholders have been considered in research design or conduct; 13, strengths and limitations critically discussed.
